# Interpretable Machine Learning Prediction of the Dielectric Constant and Bandgap of Polymers for Flexible Electronics

**DOI:** 10.3390/polym18182182

**Published:** 2026-09-08

**Authors:** Fatih Gül

**Affiliations:** Artificial Intelligence and Internet of Things Research Laboratory, Department of Electrical-Electronics Engineering, Recep Tayyip Erdoğan University, Rize 53100, Türkiye; fatih.gul@erdogan.edu.tr

**Keywords:** polymer informatics, dielectric constant, bandgap, machine learning, flexible electronics, interpretable machine learning, SHAP, high-throughput screening

## Abstract

Polymer dielectrics are central to flexible and printed electronics, where a material must combine a sufficiently high dielectric constant with a wide electronic bandgap to suppress leakage. Experimental or first-principles screening is slow, motivating data-driven surrogates. Here we develop an interpretable machine learning workflow that predicts both the total dielectric constant and the HSE bandgap of polymer repeat units directly from a monomer structure, using an open density-functional-theory dataset of 284 four-block polymers. Polymers are encoded with 217 RDKit descriptors and a 1024-bit Morgan fingerprint; four regressors are benchmarked under nested five-fold cross-validation with paired significance testing. The bandgap reaches $R^{2}=0.83\pm 0.04$ (MAE =0.35 eV) and the total dielectric constant $R^{2}=0.64\pm 0.06$ (MAE =0.44), but paired tests find the models statistically indistinguishable for the bandgap. Decomposing the permittivity explains its lower ceiling: the ionic component is only 15% of the magnitude yet carries 28% of the squared error, and learning curves confirm a representational rather than a data-quantity limit. Read through the Penn relation, the SHAP descriptors yield an explicit design rule—raise permittivity with polar, non-conjugated motifs rather than extended conjugation. Screening 571 unseen candidates with bootstrap uncertainties, an applicability domain and a threshold sensitivity analysis nominates carbamate/urea-type wide-bandgap high-*k* repeat units.

## 1. Introduction

Flexible, printed and wearable electronics rely on polymer dielectrics as gate insulators, capacitor layers and encapsulants [1,2]. A useful dielectric polymer must simultaneously (i) offer a high enough dielectric constant (permittivity) for charge storage or gate coupling and (ii) retain a wide electronic bandgap so that leakage current and dielectric breakdown remain low [3,4]. These requirements are physically in tension: across chemical space, the dielectric constant and the bandgap tend to vary inversely, because the same polarizable, conjugated or heteroatom-rich motifs that raise the permittivity also narrow the gap [3]. Identifying repeat units that balance the two is therefore a genuine multi-objective design problem rather than a single-property optimization.

The bandgap enters this problem as a physically motivated proxy rather than as an end in itself. In organic dielectrics the gap controls the onset of electronic conduction and thus the leakage current, the dielectric loss at a high field and—indirectly—the breakdown strength, so a wide gap is a prerequisite for a usable insulator, regardless of how attractive its permittivity is [3,4]. This is why high-throughput polymer-dielectric studies routinely screen on the (ε, $E_{g}$) pair rather than on ε alone [5,6].

Exploring this space experimentally is slow and costly, and even first-principles (DFT/HSE) evaluation of each candidate is computationally expensive [5,6]. Data-driven surrogate models trained on existing computed data are therefore attractive: once trained, they predict properties for thousands of hypothetical polymers in seconds, and—if interpretable—reveal which structural motifs control each property [7,8,9,10].

Polymer informatics has advanced rapidly along two complementary directions. The first is representation learning: graph neural networks that operate directly on the molecular graph or on ensembles of stochastic repeat units [11,12,13], and chemical language models such as TransPolymer and polyBERT that learn fingerprints from tens of millions of hypothetical polymer SMILES [14,15]. These models set the accuracy ceiling for large, multi-property datasets and, in the case of polyBERT, reduce fingerprinting cost by two orders of magnitude [15]. The second is curated platform building: the Polymer Genome service [16,17], the PI1M benchmark library [18] and the open DFT polymer datasets on which most of this work rests [5,6]. Alongside these, descriptor-based interpretable models remain actively used where the design question is *which chemistry to change* rather than *what the next decimal of accuracy is*, for polyimides [19], epoxy resins [20], general structure–property modeling [21] and physically grounded monomer design [22].

**Figure 1:**
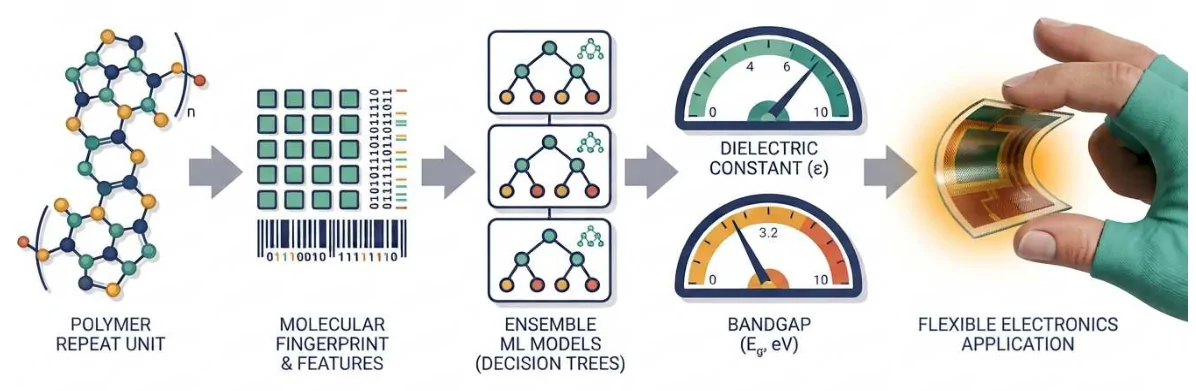
Overview of the interpretable machine learning workflow. A polymer repeat unit (SMILES) is featurized with 217 RDKit descriptors and a 1024-bit Morgan fingerprint; an ensemble of regressors is trained and benchmarked under nested five-fold cross-validation; both the dielectric constant (ε) and the bandgap ($E_{g}$) are predicted directly from the structure; candidate repeat units are screened to identify wide-bandgap, high-*k* polymers for flexible-electronics applications.

The present work belongs to that interpretable strand, and its novelty lies in the completeness of the decision chain rather than in a new architecture. Deep graph and language models remain the reference for raw accuracy on large corpora—we quantify that comparison in Section 3.3—but they do not answer the question a materials designer actually faces: *which* chemical change moves which property, by how much, and how far can the resulting recommendation be trusted. We contribute a complete, auditable and fully reproducible answer to that question for a specific design target—wide-bandgap, high-*k* repeat units for flexible electronics—in which every step is exposed: (1) *both* the total dielectric constant and the HSE bandgap are predicted from monomer SMILES with a single shared representation; (2) four standard regressors are benchmarked under nested cross-validation, and their differences are subjected to paired significance testing rather than reported as a ranking; (3) the governing descriptors are exposed via SHAP and interpreted against polymer electronic structure; (4) the difficulty of the dielectric constant is diagnosed by decomposing it into its electronic and ionic parts; (5) predictions carry bootstrap uncertainties and an explicit applicability domain; and (6) an open candidate pool is screened with a threshold sensitivity analysis and cross-checked against an independent model distributed with the same data. Points (2), (4), (5) and (6) are, to our knowledge, reported here for the first time for this system, and together, they are what turns a property regression into a screening protocol whose reliability and failure modes are quantified rather than assumed. The electronic/ionic decomposition in particular converts a known qualitative statement—that the ionic response is hard to learn—into a measured error budget, and it is what yields the physically grounded design rule of Section 3.7. Figure 1 summarizes the workflow.

## 2. Materials and Methods

### 2.1. Dataset

We use the openly available four-block polymer dataset of Mannodi-Kanakkithodi et al. [5], comprising 284 polymer repeat units built from combinations of seven building blocks, each with DFT-computed electronic, ionic and total dielectric constants and an HSE06 bandgap. Only the SMILES string, total dielectric constant (range 2.6–10.7) and HSE bandgap (0.9–8.6 eV) are used as inputs and targets. One entry with an invalid SMILES was removed, leaving 284 polymers.

### 2.2. Chemical Coverage and Representativeness of the Dataset

Because the size and composition of the dataset bound everything that follows, we state its coverage explicitly. The 284 repeat units are all four-block sequences drawn from the seven blocks ${\mathrm{CH}}_{2}$, CO, NH, O, CS, $C_{6}H_{4}$ and $C_{4}H_{2}$S, which appear in 194, 140, 138, 137, 138, 196 and 193 of the entries, respectively; the blocks are therefore close to evenly represented, and no single chemistry dominates. This space spans the saturated–aliphatic to conjugated–aromatic range relevant to organic dielectrics, and it contains recognizable real polymers as special cases—polyethylene ${\left({\mathrm{CH}}_{2}\right)}_{4}$, polyoxymethylene, poly(*p*-xylylene) (parylene N), poly(phenylene oxide), an ether-ketone (PEEK-type) sequence, polyureas, poly(*p*-phenylene) and polythiophene—which we use as an internal benchmark in Section 3.12. The targets cover ε=2.6–10.7 and $E_{g}=0.9$–8.6 eV, i.e., the full range from a low-*k* wide-gap insulator to a narrow-gap conjugated semiconductor.

The space is equally clear about what it excludes. Polymers requiring fluorine, pendant methyl or phenyl side groups, naphthalene units, ring-opened or stereo-defined backbones—PVDF, PMMA, PS, PET and PEN among them—cannot be written as four-block sequences over this block set at all, so the models are not applicable to them, and we do not report predictions for them. Copolymers, tacticity, molecular weight and chain-length effects are likewise absent by construction. This is the principal limitation of the underlying data, and we quantify its consequences in Section 3.4 and Section 3.9, and discuss them in Section 3.13.

### 2.3. Molecular Representation

Each SMILES was parsed with RDKit [23] and encoded by (i) the full set of 217 RDKit two-dimensional molecular descriptors (constitutional, topological, electrotopological and surface-area terms) and (ii) a 1024-bit radius-2 Morgan (ECFP4) fingerprint [24], concatenated into a 1241-dimensional feature vector. Non-finite descriptor values were median-imputed.

### 2.4. Models and Evaluation

Four regressors were compared: ridge regression (linear baseline, on descriptors), random forest [25], XGBoost [26] and LightGBM [27] (all on the combined descriptor+fingerprint vector), implemented with scikit-learn [28]. Performance was estimated by nested five-fold cross-validation: an outer five-fold loop gives an unbiased estimate, while an inner three-fold grid search selects hyperparameters within each outer training split, preventing information leakage. We report the coefficient of determination $R^{2}$, mean absolute error (MAE) and root-mean-square error (RMSE) as the mean ± standard deviation over the outer folds, together with parity plots from the out-of-fold predictions.

The searched hyperparameter grids are listed in Table 1. All splits, bootstrap resamples and model initializations are driven by a single fixed seed (42), so the entire manuscript—including every number, table and figure—is regenerated exactly by running the released scripts; the software versions used are given in Section 2.9.

### 2.5. Statistical Comparison of Models

Differences between models of a few hundredths in $R^{2}$ on 284 samples are not self-evidently meaningful, so every pairwise comparison is tested. For each pair we apply (i) a two-sided Wilcoxon signed-rank test on the paired per-molecule absolute out-of-fold errors (n=284), which makes no distributional assumption, and (ii) a paired *t*-test on the five outer-fold $R^{2}$ values, which is the comparison most readers make implicitly. We treat a model as preferable only when the difference survives these tests, and otherwise report the models as statistically indistinguishable.

### 2.6. Uncertainty Quantification and Applicability Domain

Point predictions alone are not actionable for screening, so each prediction carries an uncertainty [29]. Within every outer fold we refit the selected model on 100 bootstrap resamples of that fold’s training split and take the ensemble spread $\sigma_{\mathrm{boot}}$; the total predictive standard deviation combines it with the out-of-fold residual scale $\sigma_{\mathrm{res}}$ as $\sigma_{\mathrm{tot}}={\left(\sigma_{\mathrm{boot}}^{2}+\sigma_{\mathrm{res}}^{2}\right)}^{1/2}$. Because the outer test molecules never enter any bootstrap fit, the empirical coverage of the resulting 95% intervals is an honest estimate.

The applicability domain is defined by chemical similarity to the training set [30]: for each molecule we compute the maximum Tanimoto similarity of its Morgan fingerprint to any training molecule, and we report prediction error as a function of that similarity. The same quantity is computed for every screened candidate, so each nomination is accompanied by both an uncertainty and a statement of how far it sits from the data the model was trained on.

### 2.7. Interpretability

For each target, a LightGBM model was fit on the descriptor block only, and TreeExplainer SHAP values [31] were computed; the mean absolute SHAP value ranks descriptor importance (Section 3.7).

### 2.8. Candidate Screening and Pool Integrity

The best model for each property was retrained on all 284 polymers and applied to the candidate repeat units of the open companion set.

The composition of that pool requires care, and the procedure used here differs from the one used in the original version of this work. The companion file lists 1087 four-block *sequences*, but a repeat unit is periodic: cyclic permutations and reversals of a sequence describe the same molecule. Comparing raw SMILES strings against the training set—as we did originally—therefore both leaves duplicate molecules in the pool and fails to detect candidates that are already present in the DFT training set. We now canonicalize every structure with RDKit before any comparison. This reduces the 1087 listed sequences to 731 distinct molecules, of which 160 are canonically identical to polymers already in the 284-molecule training set and are removed, leaving 571 genuinely unseen candidates. All screening results reported below refer to this corrected pool; the consequences of the correction are stated explicitly in Section 3.10.

A material is nominated when its predicted bandgap is at least $E_{g}^{\min}$ (insulating) and its predicted total dielectric constant is highest, i.e., the best high-*k* point of the insulating region.

The choice of $E_{g}^{\min}$ deserves justification rather than assertion. The bandgap enters as a proxy for leakage and loss, and no single value separates “insulating” from “leaky” for every application [3,4]; we therefore adopt $E_{g}^{\min}=4$ eV as the primary criterion—it sits at the 79th percentile of the training distribution (the 75th and 90th percentiles are 3.90 and 4.47 eV, and 59 of the 284 training polymers satisfy it), so it selects the wide-gap upper quintile rather than the bulk of the data—and we report in Section 3.10 how the nominated set changes for $E_{g}^{\min}\in \left\{3.0,3.5,4.0,4.5,5.0\right\}$ eV. We additionally report a risk-averse variant in which a candidate must satisfy the criterion at the lower 95% bound of its predicted gap, $E_{g}-1.96\;\sigma_{\mathrm{tot}}\geq E_{g}^{\min}$, rather than at its point prediction.

The companion candidate file also carries property values from the sequential-learning model distributed with that dataset [5]. These are predictions of an independently trained model, not new DFT calculations, so they cannot serve as the ground truth; we nevertheless use them as an independent cross-check on our own predictions (Section 3.11).

### 2.9. Software and Reproducibility

All computations were run in Python 3.13.5 with RDKit 2026.03.5, scikit-learn 1.8.0, XGBoost 3.3.0, LightGBM 4.7.0, SHAP 0.52.0, SciPy 1.15.3, NumPy 2.2.6 and pandas 2.2.3, on a single desktop workstation; the complete nested cross-validation of all four models and both properties takes approximately two minutes of wall-clock time, and the full revision analysis suite (bootstrap intervals, learning curves, ablations) takes approximately one hour. The dataset, the analysis scripts and the derived result files are provided as Supplementary Material, and every reported number carries the fixed seed described above.

### 2.10. Preparation of the Schematic Figure

Figure 1 is a conceptual schematic of the workflow and contains no measured, simulated or predicted data. Its initial version was rendered with the generative AI tool Google Gemini (Gemini 3.1 Flash Image ‘Nano Banana 2’; Google LLC, Mountain View, CA, USA; accessed on 3 July 2026) and was subsequently corrected, redrawn in part and finalized by the author, who verified that every element of the figure corresponds to the methodology described above. No generative AI was used to produce, alter or interpret any data, model, figure or result reported in this work, and every other figure in this paper was generated directly by the analysis code released with it.

## 3. Results and Discussion

### 3.1. Predictive Performance

**Figure 2:**
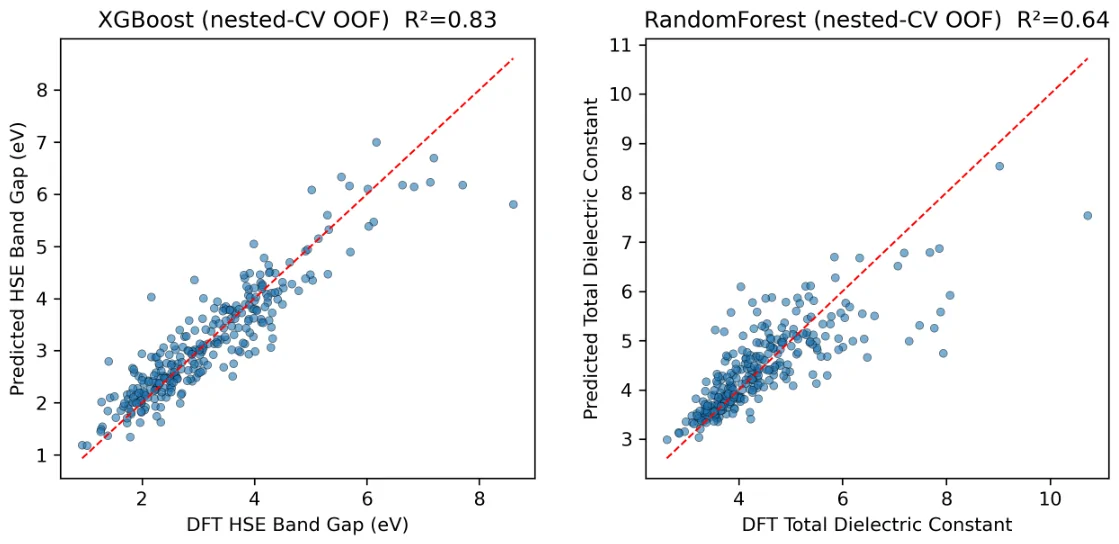
Nested cross-validation out-of-fold parity plots for the best model of each property: (**left**) HSE bandgap (XGBoost); (**right**) total dielectric constant (random forest). The dashed line is the ideal y=x.

Table 2 summarizes nested-CV performance. The bandgap is learned well by all models, with XGBoost giving the best point estimate ($R^{2}=0.834\pm 0.040$, MAE =0.349 eV, RMSE =0.476 eV); even the linear baseline reaches $R^{2}=0.81$, indicating a largely additive structure–bandgap relationship. The total dielectric constant is intrinsically harder—its ionic part depends on vibrational response only weakly encoded in a two-dimensional structure, as quantified in Section 3.6—so the best model (random forest, $R^{2}=0.639\pm 0.056$, MAE =0.439) is more modest but still useful for ranking. Parity plots (Figure 2) show tight bandgap correlation and a wider, unbiased dielectric scatter.

### 3.2. Are the Models Actually Different? Paired Significance Testing

Differences of a few hundredths in $R^{2}$ invite over-interpretation, so we test every pair as described in Section 2.5; the results are collected in Table 3, and they change how the comparison should be read.

For the total dielectric constant, all three nonlinear models beat the linear baseline by a clear margin on the per-molecule test (Wilcoxon $p=4\times 10^{-4}$, $2\times 10^{-5}$ and $8\times 10^{-4}$ for random forest, XGBoost and LightGBM against ridge), so the nonlinearity is real. Among the three nonlinear models, however, no pair is significantly different (Wilcoxon p≥0.09; paired *t*-test on fold $R^{2}$, p≥0.15): the apparent 0.640 versus 0.625 versus 0.599 ordering is within noise.

For the bandgap the conclusion is stronger still: *no* pair of models differs significantly under the paired *t*-test (p≥0.09 throughout), including ridge regression against XGBoost (p=0.090). Only the random forest–XGBoost pair reaches nominal significance on the per-molecule Wilcoxon test (p=0.024), and that single result would not survive a multiplicity correction across the six comparisons.

We therefore do not claim that XGBoost is “the best model” for the bandgap; the honest statement is that a 217-descriptor linear model, two tree ensembles and gradient boosting all extract essentially the same signal from this dataset, and that the choice among them should be made on grounds other than accuracy (interpretability, cost, or the availability of a variance estimate). We retain XGBoost for the bandgap and random forest for the dielectric constant as the models with the best point estimates, and we carry their uncertainty through the screening rather than treating them as uniquely correct.

### 3.3. Comparison with Previously Reported Models

Two comparisons are relevant: with the model originally trained on this same dataset, and with the modern deep architectures that define the current state of the art.

**Same data, earlier model.** Mannodi-Kanakkithodi et al. applied kernel ridge regression with a Gaussian kernel to these 284 polymers using a single 250/34 train/test split, and reported an average error “of the order of 10% or less” across the three dielectric components and the bandgap [5]. Our out-of-fold errors correspond to a mean absolute error of 9.9% of the mean value for the total dielectric constant (MAPE 9.2%) and 11.1% for the bandgap (MAPE 12.0%). The two approaches are therefore of equivalent accuracy, with the important qualification that our figure is obtained under nested five-fold cross-validation—in which hyperparameters are selected *inside* each training split and every one of the 284 polymers is predicted exactly once by a model that never saw it—rather than from a single held-out subset of 34 molecules. The comparison establishes that the interpretable pipeline reported here pays no accuracy penalty for its transparency: the SHAP analysis of Section 3.7 therefore describes a model that is competitive on this dataset, not a weakened stand-in adopted for the sake of interpretability.

**Deep architectures.** Graph neural networks and chemical language models occupy a different operating regime. polyBERT is trained on 80 million hypothetical polymer structures and mapped to properties through multitask learning [15]; polyGNN and related multitask graph models are trained on tens of thousands of labeled polymer–property pairs [12,13]; TransPolymer likewise relies on large-scale pretraining [14]. Their advantage comes from exactly what is missing here—data volume and learned representations—and we would expect them to outperform descriptor models where such data exist. We did not retrain them on these 284 points, and we would caution against reading any such comparison as meaningful: a model with millions of parameters evaluated on 284 samples measures the pretraining corpus, not the architecture. The learning curves in Section 3.4 make the same point from the other direction, showing that, on this dataset, the accuracy is limited by representation and label physics rather than by model capacity. The appropriate division of labor is that large pretrained models supply broad screening across chemical space, while compact interpretable models of the kind used here supply auditable decision rules within a defined chemical family—which is why descriptor-based interpretable modeling remains in active use for polyimides [19], epoxies [20] and monomer design [22].

### 3.4. How Much Is the Small Dataset Costing Us? Learning Curves

**Figure 3:**
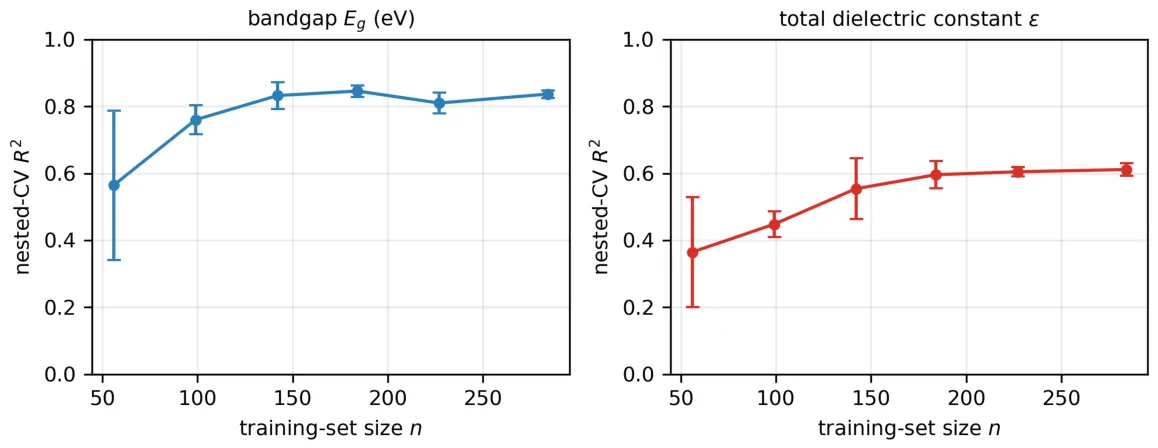
Learning curves for (**left**) the HSE bandgap and (**right**) the total dielectric constant. Points are means over three repetitions of the full nested cross-validation on random subsets; error bars are standard deviations. Both curves plateau before the full dataset is used.

A dataset of 284 molecules invites the concern that the models are unstable or overfitted and that the reported accuracy is an artifact of the sample size. We address this directly by repeating the entire nested cross-validation on random subsets of increasing size, with three independent repetitions at each size and a fresh fold assignment in each repetition (Figure 3, Table 4).

The curves settle well before the full dataset is reached. For the bandgap, $R^{2}$ rises from 0.56±0.22 at n=56 to 0.83±0.04 at n=142 and then oscillates between 0.81 and 0.85: half the data already suffices, and the last 142 molecules add nothing detectable. For the total dielectric constant the approach to the plateau is slower but equally clear—0.36→0.55→0.60→0.61 at n=56, 142, 227 and 284—with the final 20% of the data worth less than 0.01 in $R^{2}$. The shrinking repetition-to-repetition spread (from ±0.16 at n=56 to ±0.02 at n=284 for the dielectric constant) also shows that the models are stable at full size rather than sample-dependent.

The practical consequence is a diagnosis rather than a caveat. Because both curves are flat at the right-hand end, the ceiling of $R^{2}\approx 0.64$ for the dielectric constant is *not* a data-quantity limit: adding more four-block polymers of the same kind would not lift it. The limitation lies in the representation and in the physics of the target—two-dimensional descriptors of an isolated repeat unit cannot resolve the vibrational response that produces the ionic part of the permittivity, as Section 3.6 demonstrates quantitatively. Progress on this target requires different *information* (vibrational or three-dimensional features, or explicit ionic-response labels), not more rows.

### 3.5. Where the Models Fail: Residual Analysis

**Figure 4:**
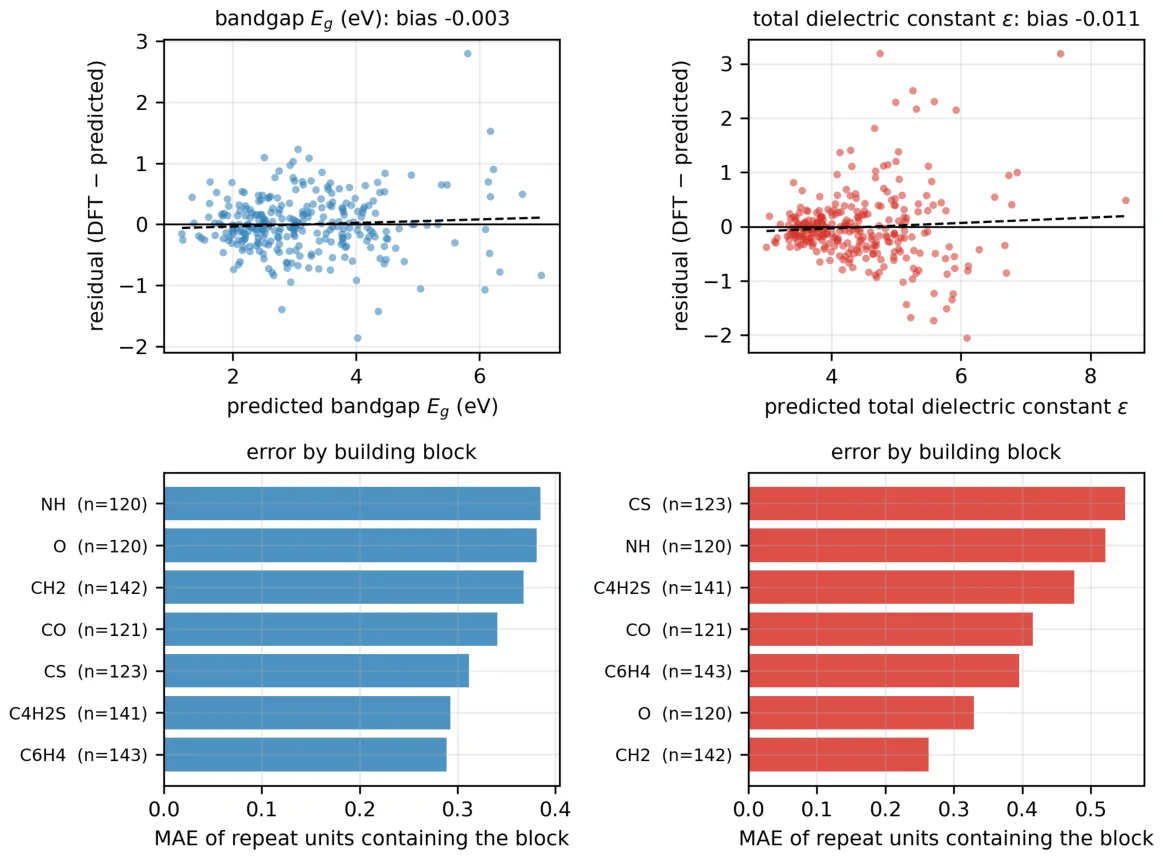
Residual diagnostics. (**Top**) out-of-fold residuals versus predicted value for the bandgap (**left**) and the total dielectric constant (**right**); dashed lines are least-squares trends. (**Bottom**) mean absolute error of the repeat units containing each building block.

Aggregate metrics hide the chemistry of the errors, so we examine the out-of-fold residuals directly (Figure 4). Both models are essentially unbiased overall (mean residual −0.003 eV for the bandgap and −0.011 for the dielectric constant), and the residual clouds are symmetric, but the errors are not uniformly distributed over chemistry.

For the *total dielectric constant*, the largest errors occur for repeat units containing thiocarbonyl (CS, MAE 0.550) and amine (NH, MAE 0.521) blocks, and the smallest for those containing methylene (${\mathrm{CH}}_{2}$, MAE 0.264) and ether oxygen (O, MAE 0.329)—a spread of more than a factor of two. This ordering is exactly what the decomposition of Section 3.6 predicts: CS and NH are the strongly polar, hydrogen-bonding blocks that generate the largest ionic contributions, and the ionic term is the component the descriptors cannot see. ${\mathrm{CH}}_{2}$-rich sequences, whose permittivity is almost purely electronic, are predicted twice as accurately.

For the *bandgap* the pattern is different and reflects the sampling of the label range: the largest errors belong to NH (0.385 eV), O (0.381 eV) and ${\mathrm{CH}}_{2}$ (0.368 eV) blocks—the saturated, wide-gap chemistries that populate the sparse upper tail of the distribution—while the conjugated $C_{6}H_{4}$ (0.289 eV) and $C_{4}H_{2}$S (0.293 eV) blocks, which dominate the well-sampled 1–4 eV region, are predicted best. The models regress wide-gap outliers toward the center of the training distribution; the extreme case is polyethylene, whose DFT gap of 8.61 eV is predicted as 5.81 eV (Section 3.12). This is a boundary effect of the label distribution, not a chemical failure, and it is precisely what the applicability-domain analysis in Section 3.9 is designed to flag before such a prediction is acted upon.

### 3.6. Why the Dielectric Constant Is Harder: Electronic/Ionic Decomposition

The original version of this work attributed the modest $R^{2}$ of the dielectric constant to its ionic contribution without testing that claim. The dataset reports the electronic and ionic components separately [5], so the claim can be tested directly, and the result is more nuanced than the original assertion.

**Figure 5:**
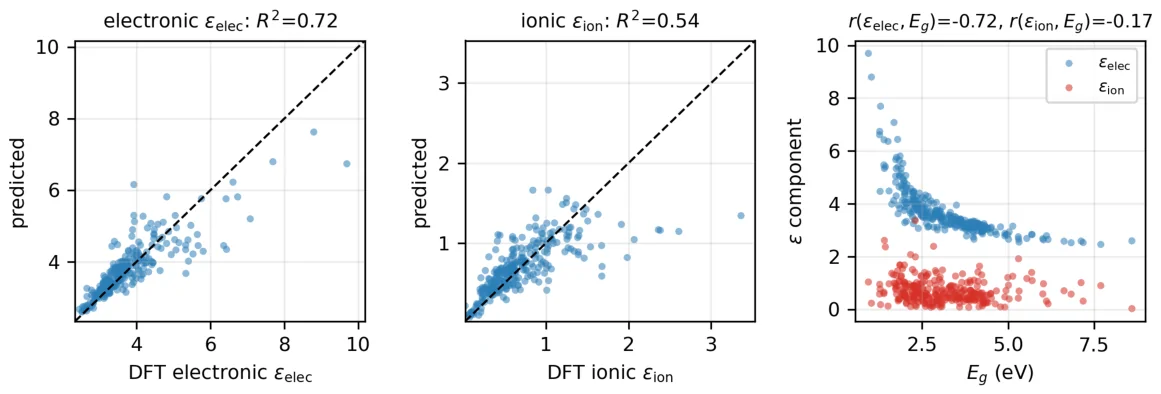
Decomposition of the permittivity. (**Left**, **center**) out-of-fold parity plots for the electronic and ionic components. (**Right**) both components against the bandgap: the electronic part follows the inverse Penn-type relation, whereas the ionic part is almost independent of the gap—the asymmetry that permits high-*k* wide-gap design. Dashed lines in the left and center panels are the ideal *y* = *x*.

**The ionic term is small but disproportionately hard.** Across the 284 polymers the ionic component accounts for a mean of 15.2% of the total permittivity (median 14.1%, 90th percentile 26.6%, maximum 46.1%), with $\varepsilon_{\mathrm{ion}}$ spanning 0.02–3.36. Training the same model separately on each component (Table 5, Figure 5) gives $R^{2}=0.710\pm 0.089$ for $\varepsilon_{\mathrm{elec}}$ but only 0.549±0.128 for $\varepsilon_{\mathrm{ion}}$. In relative terms the gap is far larger than these $R^{2}$ values suggest: the mean relative error is 7.4% for the electronic component and 32.8% for the ionic one. The ionic term thus carries 27.6% of the total squared error while contributing only 15.2% of the magnitude—roughly 1.8× its proportional share.

**But the electronic term still dominates the absolute error.** Two oracle experiments make the budget explicit. Replacing the predicted ionic component with its true value raises $R^{2}$ on the total from 0.645 to 0.796; replacing the predicted *electronic* component with its true value raises it to 0.922. Because $\varepsilon_{\mathrm{elec}}$ has more than twice the standard deviation of $\varepsilon_{\mathrm{ion}}$ (0.95 versus 0.46), its residual error (MAE 0.307) exceeds the ionic one (MAE 0.193), even though it is predicted far more accurately in relative terms. The correct statement is therefore that the ionic response is the intrinsically harder *physics*—it depends on infrared-active vibrational modes and their effective charges, which a two-dimensional descriptor of an isolated repeat unit does not encode—while the electronic response, though well captured, sets the absolute error scale simply because it is the larger and more variable quantity. We have corrected this manuscript accordingly.

**Decomposing does not help.** Predicting the two components separately and summing them gives $R^{2}=0.637$ on the total, statistically identical to predicting the total directly (0.645). The decomposition is therefore valuable as a diagnosis, not as a modeling strategy: it identifies where the missing information lies (vibrational response) rather than recovering it. Together with the flat learning curve of Section 3.4, this localizes the $R^{2}\approx 0.64$ ceiling precisely—it is a consequence of the descriptors and of the target physics, and neither more data of the same kind nor a better regressor will remove it. Vibrational or three-dimensional descriptors, or explicit $\varepsilon_{\mathrm{ion}}$ labels used as an auxiliary task, are the routes that would.

### 3.7. Interpretability (SHAP) and Its Physical Basis

**Figure 6:**
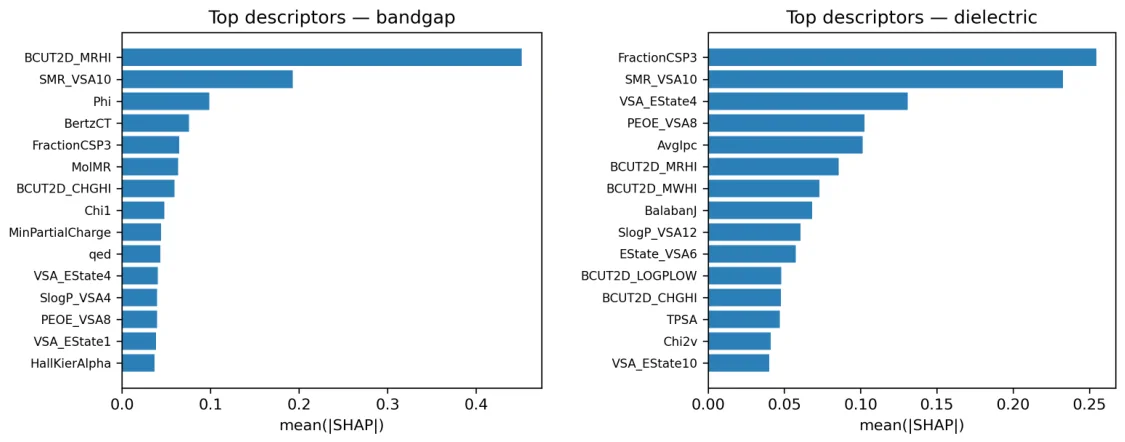
Top-15 descriptors by mean absolute SHAP value for (**left**) bandgap and (**right**) total dielectric constant.

For the bandgap, the dominant descriptors are an atomic-property–weighted connectivity eigenvalue tied to electronic structure (BCUT2D_MRHI), molecular-complexity terms (BertzCT, Kier Φ) and the ${\mathrm{sp}}^{3}$ fraction (FractionCSP3). For the total dielectric constant, the leading descriptors are FractionCSP3 and surface-area terms weighted by molar refractivity and partial charge (SMR_VSA10, VSA_EState4, PEOE_VSA8), i.e., polarizable and heteroatom-rich surface area raises permittivity (Figure 6).

These rankings are not merely plausible; they follow from the electronic structure of the repeat unit, and it is worth setting out why, because the mechanism also explains the trade-off the screening has to negotiate.

**Why polarizability descriptors govern both properties.** In the Penn single-oscillator picture the electronic permittivity of a semiconductor obeys $\varepsilon_{\infty }≃1+{\left(\hbar \omega_{p}/E_{g}\right)}^{2}$, with $\omega_{p}$ the valence plasma frequency [32]; the closely related Moss rule expresses the same inverse coupling between the refractive index and gap [33]. Electronic polarizability and bandgap are therefore two readings of the same underlying quantity—the energy scale of valence excitations—and any descriptor that tracks polarizability must appear, with opposite sign, in both models. BCUT2D_MRHI is exactly such a descriptor: it is the largest eigenvalue of the Burden connectivity matrix weighted by atomic molar refractivity, and molar refractivity is proportional to electronic polarizability through the Lorentz–Lorenz relation. Its dominance in the bandgap model is thus the Penn relation expressed in graph-theoretic form: the more polarizable the most strongly bonded environment, the narrower the gap. SMR_VSA10—the van der Waals surface area carried by atoms in the highest molar-refractivity bin, in this chemical space, essentially sulfur and aromatic carbon—plays the same role from the surface side and, consistently, ranks in the top five for *both* targets.

**Why ${\mathrm{sp}}^{3}$ fraction is the strongest lever.** FractionCSP3 measures saturation, i.e., the absence of π conjugation. Saturated aliphatic sequences have deep, narrow valence bands, and hence wide gaps and low electronic polarizability; extended conjugation ($C_{6}H_{4}$, $C_{4}H_{2}$S blocks) delocalizes the π system, raises $\varepsilon_{\mathrm{elec}}$ and collapses the gap. That single structural axis therefore moves both properties, in opposite directions—which is precisely the physical origin of the inverse trade-off visible in Section 3.10, and the reason that simply maximizing ε produces useless (conducting) candidates.

**Which descriptors break the trade-off?** The remaining dielectric-specific descriptors—VSA_EState4 and PEOE_VSA8, surface areas binned by electrotopological state and by partial charge—respond to polar heteroatom groups such as C=O and N–H rather than to conjugation. Polar bonds of this kind raise the permittivity through the *ionic* (infrared-active vibrational) channel, which, unlike the electronic channel, is not tied to the gap by the Penn relation: in this dataset $\varepsilon_{\mathrm{elec}}$ correlates strongly and negatively with $E_{g}$ (Pearson r=−0.72, Spearman −0.91), whereas $\varepsilon_{\mathrm{ion}}$ is only weakly related to it (r=−0.17, Spearman −0.20; Section 3.6). This asymmetry is the actionable design rule that comes out of the interpretability analysis: *to raise permittivity without closing the gap, add polar but non-conjugated functionality—carbonyl, urea and carbamate motifs—rather than extending the conjugated backbone.* The screening in Section 3.10 converges on exactly such carbonyl/urea-rich sequences, so the SHAP analysis, the physics and the nominations tell a single consistent story rather than three separate ones.

### 3.8. Feature Redundancy and Representation Ablation

The 1241-dimensional vector concatenates 217 descriptors with a 1024-bit fingerprint without any selection step, and it is reasonable to ask what that redundancy costs. We quantify it in three ways.

**How redundant is it?** Of the 217 RDKit descriptors, 68 are constant across this dataset—they encode chemistry (charged species, stereocenters, uncommon heteroatoms) that the seven-block space never realizes—and are therefore uninformative by construction. Among the remaining descriptors only 216 of the 23,436 pairs exceed |r|>0.95 (1568 exceed |r|>0.90), so severe collinearity is confined to a small number of families (surface-area and connectivity variants). On the fingerprint side, 425 of the 1024 bits are ever set; the remaining 599 are constant zeros.

**Does it matter?** Table 6 reports the full nested cross-validation for five representations. Greedily pruning descriptors to |r|≤0.95 removes 35 of them and changes $R^{2}$ by at most 0.003 for either target. Descriptors alone are nearly as good as the combined vector (0.631 vs. 0.640 for the dielectric constant, 0.829 vs. 0.834 for the bandgap), while the fingerprint alone is clearly weaker for the dielectric constant (0.577). The concatenation is thus mildly beneficial and not harmful: tree ensembles are insensitive to duplicated and constant inputs, which is why we retain the full vector for the reported results.

**What this implies.** The redundancy is real but benign, and a 183-descriptor pruned representation would reproduce every conclusion of this paper at 15% of the feature count. More importantly, the ablation shows that the accuracy ceiling is not caused by feature dilution: no reduction of the input space improves either target, which is consistent with the diagnosis in Section 3.4 and Section 3.6 that the limitation lies in the physics the descriptors omit rather than in the descriptors they include.

### 3.9. Uncertainty and Applicability Domain

Section 2.6 described how each prediction acquires an uncertainty and a chemical-similarity score. Their behavior here is instructive, and in one respect, it contradicts the assumption usually made about applicability domains.

**Figure 7:**
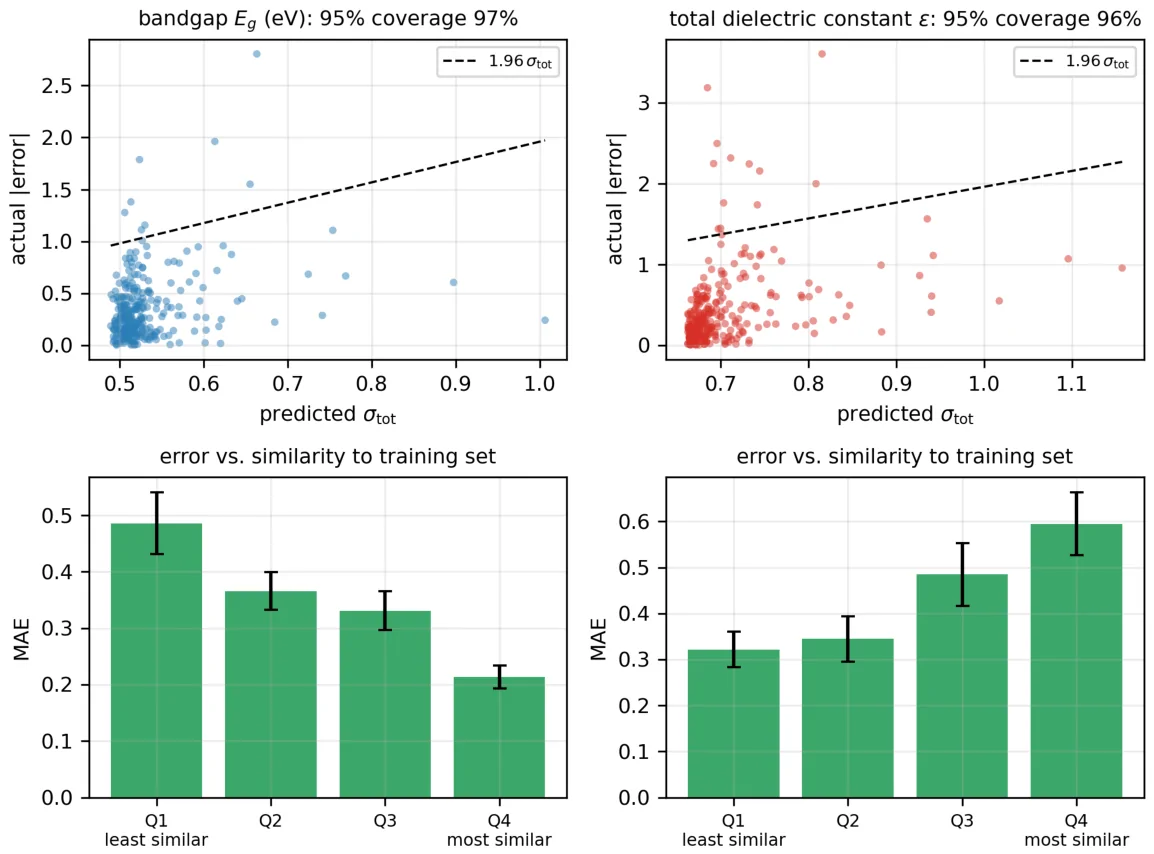
Uncertainty and applicability domain. (**Top**) realized absolute error against the calibrated predictive standard deviation; the dashed line is the nominal 95% bound. (**Bottom**) mean absolute error by quartile of maximum Tanimoto similarity to the training set; error bars are standard errors. The bandgap follows the expected trend; the dielectric constant inverts it because similarity is confounded with the target magnitude.

**Bootstrap intervals need calibration, and then work.** The bootstrap spread alone is far too narrow: nominal 95% intervals built from the ensemble scatter cover only 54% (dielectric constant) and 62% (bandgap) of the true values, because resampling captures model variance but not the irreducible error. Combining it with the out-of-fold residual scale as described in Section 2.6 restores honest calibration—95.8% and 97.2% empirical coverage against a 95% nominal level—and the resulting $\sigma_{\mathrm{tot}}$ is genuinely informative, correlating with the realized absolute error at Spearman ρ=0.50 (dielectric constant) and 0.22 (bandgap) (Figure 7, top row).

**Chemical similarity is a good domain indicator for the bandgap and a misleading one for the permittivity.** Sorting the training molecules by their maximum Tanimoto similarity to the rest of the set, the bandgap behaves as expected—the MAE falls monotonically from 0.486 eV in the least-similar quartile to 0.213 eV in the most-similar one. For the dielectric constant the trend *reverses*: the MAE rises from 0.322 to 0.595 as similarity increases (Figure 7, bottom row). This is not a failure of the model but a confound of the chemical space. Similarity is strongly correlated with the property itself (Spearman +0.46 with ε, −0.56 with $E_{g}$), because the most “typical” members of this combinatorial space are the aromatic and thiophene-rich sequences, which are precisely the high-permittivity, narrow-gap ones; the dielectric error scales with the magnitude of the target (Spearman +0.40). Once expressed relatively, the dielectric error is almost flat across quartiles (8.1%, 7.4%, 10.0%, 11.3%).

The practical conclusion is that the two reliability signals are complementary rather than interchangeable, and that reporting only one would be misleading. For the bandgap, similarity to the training set is the better guide—which is also why the wide-gap, aliphatic outliers such as polyethylene (Section 3.12) are correctly flagged as unreliable. For the dielectric constant, the bootstrap $\sigma_{\mathrm{tot}}$ is the better guide, and similarity should be used only after normalizing for the predicted magnitude. Both are reported for every screened candidate in Section 3.10.

### 3.10. Screening for Flexible-Electronics Dielectrics

**A correction to the candidate pool.** Applying the canonicalization described in Section 2.8 changes the composition of the screening pool materially, and we report the change rather than only its result. The 1087 listed four-block sequences correspond to 731 distinct molecules—356 of the listed rows are cyclic permutations or reversals of another row—and 160 of those molecules are canonically identical to polymers already in the DFT training set. The original version of this work screened the raw list and consequently included training molecules among its nominations: the polymer reported there as the top candidate, CO–O–CO–NH, is the same molecule as the training entry NH–CO–O–CO, whose DFT values are ε=4.57 and $E_{g}=5.31$ eV. Its apparently excellent predicted values (ε=4.55, $E_{g}=5.30$ eV) were therefore largely memorization. All results below use the corrected pool of 571 genuinely unseen molecules. The cross-validation results are unaffected, since the 284 training polymers are all canonically distinct.

**Figure 8:**
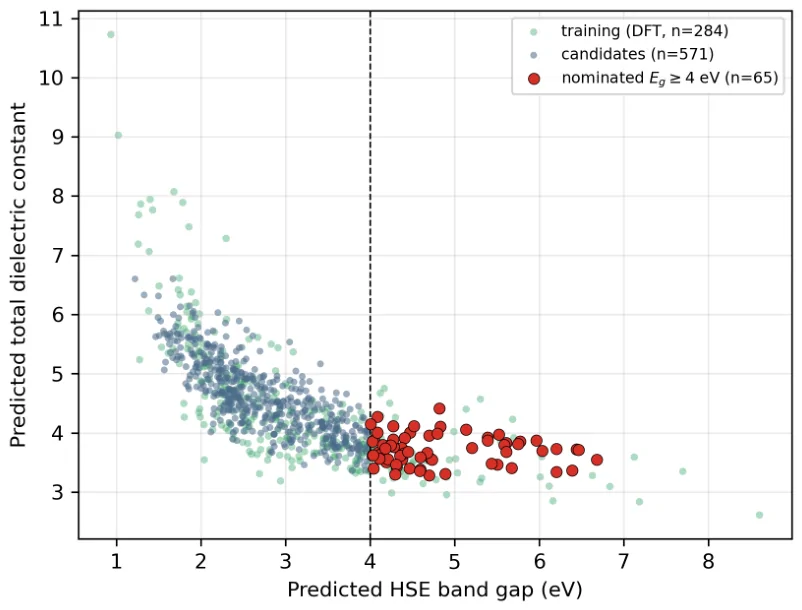
Screening of the corrected candidate pool: predicted total dielectric constant versus predicted bandgap, showing the inverse trade-off. Training data (DFT, green), the 571 deduplicated candidates (blue), and nominated wide-bandgap high-*k* polymers (red, $E_{g}\geq 4$ eV, dashed line).

**Figure 9:**
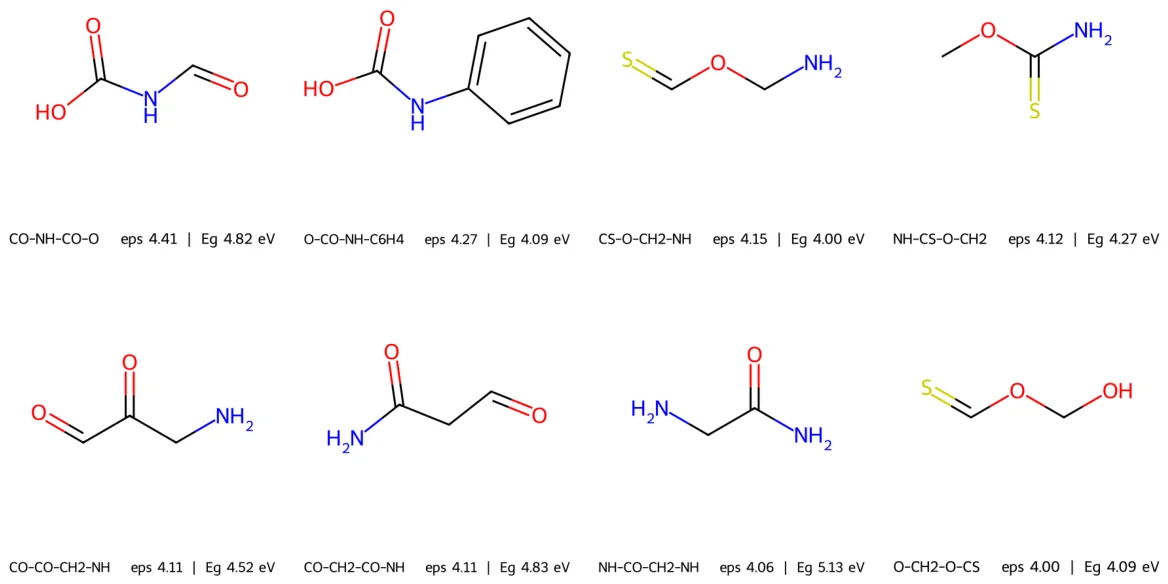
Repeat-unit structures of the highest-ranked insulating candidates, with their predicted total dielectric constant and bandgap. Atoms are colored by element following the standard RDKit scheme: red, oxygen; blue, nitrogen; yellow, sulfur; black, carbon.

**The screening landscape.** Applying the models to the corrected pool reproduces the expected inverse bandgap–permittivity trade-off (Figure 8). Of the 571 candidates, 65 are insulating at the $E_{g}\geq 4$ eV criterion, and 19 remain when the criterion is applied to the lower 95% bound of the predicted gap. The highest-permittivity insulating repeat units are carbonyl-rich carbamate, urea and imide-type motifs, exactly the chemistry that the SHAP analysis of Section 3.7 predicts should raise permittivity without closing the gap—polar functionality without extended conjugation. The leading nomination is CO–NH–CO–O (ε=4.41±0.68, $E_{g}=4.82\pm 0.63$ eV), followed by O–CO–NH–$C_{6}H_{4}$ (4.27±0.67, 4.09±0.50 eV) and CS–O–${\mathrm{CH}}_{2}$–NH (4.15±0.69, 4.00±0.54 eV); Table 7 lists the ten best with their uncertainties and Figure 9 shows their structures.

Two qualifications belong with these numbers. First, the predictive standard deviations are not small relative to the spread of the nominated set ($\sigma_{\varepsilon }\approx 0.67$–0.70 against a range of 3.9–4.4 in ε), so the *ordering* within the top ten should not be taken literally; what the screening supports is the identification of a chemically coherent family, not a ranking of individual sequences. Second, the nominated candidates sit at moderate chemical distance from the training data (maximum Tanimoto similarity 0.29–0.71, against a pool mean of 0.57), which places them inside the region where Section 3.9 found the bandgap model to be least reliable. Both facts argue for treating the nominations as a shortlist for first-principles follow-up rather than as design conclusions.

**Sensitivity to the threshold.** Because the $E_{g}\geq 4$ eV criterion is a design choice rather than a physical constant, Table 8 reports the nominated set for thresholds from 3 to 5 eV, and additionally under the risk-averse rule that the criterion must hold at the lower 95% bound of the predicted gap. The nominated set contracts smoothly and without discontinuity—246, 157, 65, 34 and 21 candidates at 3.0, 3.5, 4.0, 4.5 and 5.0 eV—and the same carbonyl/urea/carbamate chemistry dominates the top of the list at every threshold, so the qualitative conclusion does not depend on the cut. What the threshold does control is the achievable permittivity: the best predicted ε falls from 5.53 at 3 eV to 4.06 at 5 eV, a direct measurement of the trade-off the design problem must negotiate. The uncertainty-aware counts are markedly smaller at every threshold (63, 32, 19, 9 and 4), which is the honest expression of how much of the nominated set survives taking the model’s own uncertainty seriously.

### 3.11. Independent Cross-Check of the Predictions

**Figure 10:**
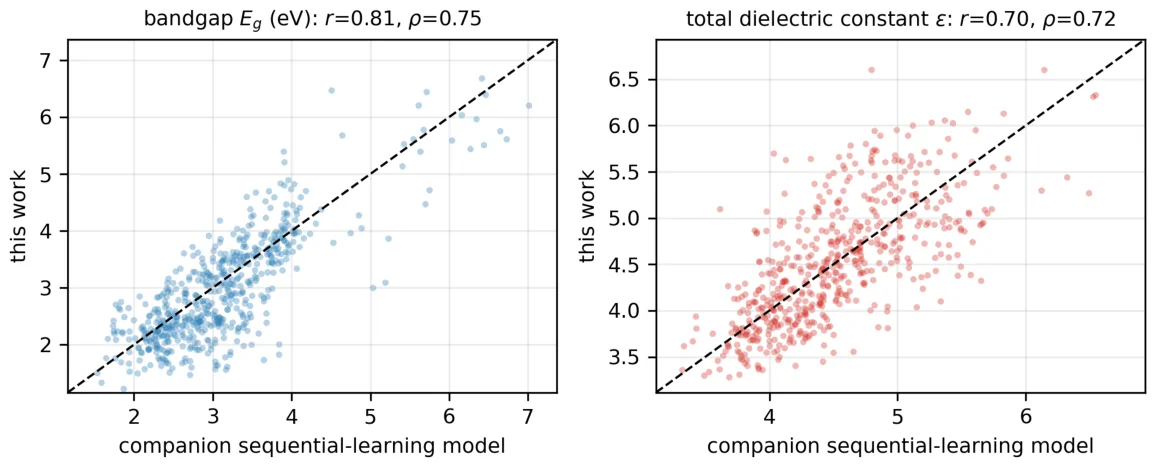
Predictions of this work against the companion sequential-learning model for the corrected candidate pool: (**left**) bandgap, (**right**) total dielectric constant. The dashed line is y=x.

The companion candidate file carries property values from the sequential-learning model distributed with the dataset [5]. These are predictions of an independently trained model—a different algorithm on a different fingerprint—not new first-principles calculations, and they cannot serve as the ground truth. They do, however, provide an external consistency check that is independent of our cross-validation. Over the 571 corrected candidates the two models agree well on the dielectric constant (Pearson r=0.695, Spearman ρ=0.719, MAE =0.374, bias =−0.006) and on the bandgap (r=0.809, ρ=0.754, MAE =0.447 eV, bias =−0.143 eV); 91% and 78% of our predictions respectively fall within one reported uncertainty of the companion value (Figure 10). The small negative bandgap bias is consistent with the shrinkage toward the distribution center identified in Section 3.5 and Section 3.12: our model is slightly more conservative than the companion model at the wide-gap end, which for a screening filter errs in the safe direction.

We emphasize what this comparison can and cannot establish. Agreement between two models trained on the same 284 DFT points does not validate either against physical reality; it establishes only that the nominations are not an artifact of our particular algorithm or descriptor choice. Genuine validation requires new HSE/DFPT calculations or synthesis and measurement, neither of which was within the capability of this study, and the nominated repeat units remain predictions until such follow-up is performed.

### 3.12. Benchmark on Chemically Recognizable Polymers

Reviewers of screening studies rightly ask how the model performs on polymers that a reader can recognize. Several of the reviewers of this manuscript specifically asked for PVDF, PMMA, PS, PET and PEN. As explained in Section 2.2, none of these can be written as a four-block sequence over the block set used here—they require fluorine, pendant methyl or phenyl groups, or naphthalene units—so any number we printed for them would be an extrapolation outside the chemical space of the training data, and we consider it more informative to say so than to report it.

The four-block space does, however, contain eleven sequences that correspond to real, named polymers. Table 9 compares, for each of them, the DFT reference value with the *out-of-fold* prediction—that is, the value produced by a model that never saw that polymer during training. Across these eleven polymers the mean absolute error is 0.27 for the dielectric constant and 0.67 eV for the bandgap, and the rank ordering is almost perfectly preserved (Spearman ρ=0.97 and 0.90 respectively). The model correctly places polyethylene and polyoxymethylene at the low-ε/wide-gap corner, the ether-ketone and urea sequences in the useful high-ε insulating region, and polythiophene alone at ε≈9 with a 1 eV gap.

The single large error is instructive. Polyethylene, the most saturated and widest-gap member of the set, is predicted at 5.81 eV against a DFT reference of 8.61 eV. This is the boundary effect identified in Section 3.5: PE sits at the extreme edge of the label distribution, with few neighbors to learn from, and the ensemble regresses it toward the mean. It is also where the difference between the DFT label and physical reality matters: the measured optical gap of polyethylene is 8.8 eV [34], in good agreement with the HSE reference but not with our prediction. Two conclusions follow. First, the model must not be used for extreme wide-gap chemistries without the uncertainty and applicability-domain checks of Section 3.9. Second, for the screening task itself, this failure mode is benign: it makes the model *conservative* at the wide-gap end, so wide-gap candidates that pass the $E_{g}\geq 4$ eV filter are, if anything, under-promoted rather than over-promoted.

Finally, a word on comparing any of these numbers with laboratory measurements. The labels are DFT values for ideal, isolated, crystalline chains at zero temperature, whereas a measured permittivity belongs to a semicrystalline film at a finite frequency and temperature, with its own morphology, free volume and moisture content; the two are not the same observable, and the discrepancy between them is a property of the physics, not of the machine learning. The magnitude of this gap for polymer dielectrics has been documented explicitly [4,34,35]. We therefore restrict quantitative validation to the DFT reference values, and treat experimental agreement as a question for the follow-up work proposed in Section 3.13.

### 3.13. Scope, Limitations and What the Model Does Not Predict

The quantities learned here are properties of an *isolated repeat unit* computed by DFT, and it is important to be explicit that this is not the same object as a processed polymer film.

**Chain-level versus solid-state structure.** The dielectric response of a real polymer depends on the aggregate structure—crystallinity, chain orientation, lamellar and amorphous fractions, free volume and density—none of which enters a monomer SMILES string. A semicrystalline film of a given chemistry can differ substantially in permittivity from the ideal crystalline chain, and orientation makes the response anisotropic, whereas the present model returns a single scalar per repeat unit. The magnitude of this effect is well documented: chemical identity, morphology and interfaces have been shown to shift the electronic structure and dielectric behavior of polymer dielectrics substantially relative to the ideal crystal [4,34,35]. Our predictions should therefore be read as a ranking over *chemistries*, to be followed by morphology-aware simulation or measurement, not as predictions of a film-level permittivity.

**Molecular weight and chain-length distribution.** Because the descriptor vector is computed from a single repeat unit, the model is, by construction, invariant to molecular weight and to its distribution: two samples of the same chemistry with different $M_{n}$ or dispersity receive identical predictions. This is acceptable in the high-molecular-weight limit, where repeat-unit properties dominate, but not for oligomers or for properties governed by chain ends. The model could be extended to include molecular weight, provided that a dataset pairing chemistry with measured $M_{n}$ and dielectric response were available; descriptors such as chain length, end-group fraction and $M_{n}$ itself would then enter alongside the structural ones. No such dataset with consistent DFT-quality labels exists for this chemical space at present, which is why we do not attempt it here.

**Voids, fillers and composites.** The workflow does not describe porous films, nanocomposites or filled systems. In those cases the measured permittivity is an effective-medium property of a two-phase system—set by filler permittivity, volume fraction, percolation and interphase regions—and the correct treatment is to use the present prediction as the *matrix* value inside an effective-medium or homogenization model, supplemented by descriptors of the filler and of the microstructure. Predicting the composite directly from a monomer SMILES string is outside the scope of this or any repeat-unit model.

**Processing history.** Thermal history, drawing, annealing, residual solvent and moisture uptake all modify the measured response and are likewise absent from the representation.

**Data-side limitations.** The 284-polymer training set is small, and the four-block space excludes entire polymer families (Section 2.2). We have therefore quantified rather than merely acknowledged the consequences: the learning curves in Section 3.4 show where performance saturates, the applicability domain analysis in Section 3.9 shows how error grows with chemical distance from the training set, and every nomination in Section 3.10 carries an uncertainty. Finally, the labels themselves are DFT values; the residual DFT-versus-experiment error—about 0.5 eV for the polyethylene bandgap, for instance (Section 3.12)—is inherited by any model trained on them, and is not reduced by better machine learning.

## 4. Conclusions

Using only open DFT data and a compact, interpretable pipeline, we predict the polymer bandgap ($R^{2}=0.83\pm 0.04$) and total dielectric constant ($R^{2}=0.64\pm 0.06$) from the monomer structure, and screen 571 candidates to nominate wide-bandgap high-*k* repeat units for flexible electronics. The accuracy matches the kernel model originally trained on this dataset under a stricter validation protocol, so the transparency costs nothing.

The more useful outcome is that the workflow now states its own limits quantitatively rather than qualitatively. Paired significance testing shows that the four regressors are statistically indistinguishable for the bandgap, so we do not claim a best algorithm. Learning curves plateau well before n=284, and decomposing the permittivity localizes the remaining error: the ionic component is only 15% of the magnitude but carries 28% of the squared error, and supplying it exactly would raise the total to $R^{2}=0.80$. The ceiling is therefore set by the physics the descriptors omit—infrared-active vibrational response—not by dataset size or model capacity, and predicting the components separately does not recover it.

Interpretation and design meet in the same place. Read through the Penn relation, the SHAP rankings say that electronic polarizability and bandgap are two readings of one quantity, while polar non-conjugated groups raise permittivity through a channel the gap does not constrain; the screening independently converges on the carbonyl-, urea- and carbamate-rich sequences this predicts. Every nomination carries a calibrated uncertainty and an applicability-domain score, and the nominated set is reported across bandgap thresholds from 3 to 5 eV, so no conclusion depends on a single arbitrary cut.

The route forward follows directly from the diagnosis: vibrational or three-dimensional descriptors, or $\varepsilon_{\mathrm{ion}}$ as an auxiliary learning task, to break the permittivity ceiling; larger pretrained graph and language models for breadth across chemical space; and first-principles or experimental follow-up on the nominated repeat units, which remain predictions until they are tested.

## Figures and Tables

**Table 1:** Hyperparameter grids searched by the inner three-fold grid search. All other parameters are library defaults. Ridge regression is fitted on the 217 descriptors; the remaining models use the combined 1241-dimensional vector.

Model	Grid
Ridge	alpha ∈{0.1,1,10,100}
Random forest	n_estimators =400; max_depth ∈{None,12}; min_samples_leaf ∈{1,2}
XGBoost	n_estimators =400; max_depth ∈{3,5}; learning_rate ∈{0.03,0.08}; subsample =0.9
LightGBM	n_estimators =400; num_leaves ∈{15,31}; learning_rate ∈{0.03,0.08}

**Table 2:** Nested five-fold cross-validation performance (mean ± standard deviation over outer folds). Best model per property in bold.

Property	Model	$R^{2}$	MAE	RMSE
Bandgap (eV)	Ridge	0.810±0.055	0.379	0.507
	Random forest	0.814±0.043	0.372	0.503
	**XGBoost**	0.834±0.040	**0.349**	**0.476**
	LightGBM	0.823±0.035	0.362	0.491
Total dielectric const.	Ridge	0.565±0.081	0.510	0.719
	**Random forest**	0.639±0.056	**0.439**	**0.657**
	XGBoost	0.610±0.092	0.429	0.676
	LightGBM	0.599±0.054	0.449	0.691

**Table 3:** Pairwise model comparison. $p_{W}$: two-sided Wilcoxon signed-rank test on paired per-molecule absolute out-of-fold errors (n=284). $p_{t}$: paired *t*-test on the five outer-fold $R^{2}$ values. Bold marks p<0.05.

Property	Comparison	MAE A	MAE B	$p_{W}$	$p_{t}$
Bandgap (eV)	Ridge vs. random forest	0.385	0.372	0.710	0.583
	Ridge vs. XGBoost	0.385	0.349	0.085	0.090
	Ridge vs. LightGBM	0.385	0.358	0.184	0.273
	Random forest vs. XGBoost	0.372	0.349	**0.024**	0.278
	Random forest vs. LightGBM	0.372	0.358	0.463	0.477
	XGBoost vs. LightGBM	0.349	0.358	0.328	0.594
Total dielectric const.	Ridge vs. random forest	0.509	0.437	**0.0004**	0.190
	Ridge vs. XGBoost	0.509	0.424	**0.00002**	0.342
	Ridge vs. LightGBM	0.509	0.447	**0.0008**	0.385
	Random forest vs. XGBoost	0.437	0.424	0.092	0.568
	Random forest vs. LightGBM	0.437	0.447	0.686	0.152
	XGBoost vs. LightGBM	0.424	0.447	0.224	0.284

**Table 4:** Learning curves: nested-CV $R^{2}$ (mean ± standard deviation over three repetitions with independent subsampling and fold assignment) as a function of training-set size.

Property	n=56	99	142	184	227	284
Bandgap (eV)	0.564±0.223	0.760±0.044	0.832±0.040	0.846±0.017	0.810±0.030	0.837±0.011
Total dielectric const.	0.365±0.164	0.448±0.038	0.554±0.090	0.596±0.041	0.605±0.014	0.612±0.019

**Table 5:** Nested-CV performance on the components of the permittivity (random forest, combined representation) and oracle experiments on the total. “Relative error” is the mean of $|y-\hat{y}|/y$. Italics mark the oracle experiments, in which one component is replaced by its true value.

Target	$R^{2}$	MAE	Relative Error	SD of Target
Electronic $\varepsilon_{\mathrm{elec}}$	0.710±0.089	0.307	7.4%	0.952
Ionic $\varepsilon_{\mathrm{ion}}$	0.549±0.128	0.193	32.8%	0.456
Total ε (direct)	0.640±0.063	0.437	9.2%	1.108
*Oracle experiments on the total*				
Predicted $\varepsilon_{\mathrm{elec}}$ + predicted $\varepsilon_{\mathrm{ion}}$	0.637	0.438		
Predicted $\varepsilon_{\mathrm{elec}}$ + *true* $\varepsilon_{\mathrm{ion}}$	0.796	0.307		
*True* $\varepsilon_{\mathrm{elec}}$ + predicted $\varepsilon_{\mathrm{ion}}$	0.922	0.193		

**Table 6:** Representation ablation under the full nested cross-validation (random forest for the dielectric constant, XGBoost for the bandgap). Pruning uses a greedy |r|≤0.95 filter on the descriptor block. Bold marks the representation used for all results reported in this work.

Representation	Dimension	$R^{2}$ (Bandgap)	$R^{2}$ (ε)
Descriptors only	217	0.829	0.631
Fingerprint only	1024	0.824	0.577
Descriptors + fingerprint (used)	1241	**0.834**	**0.640**
Pruned descriptors (|r|≤0.95)	182	0.831	0.632
Pruned descriptors + fingerprint	1206	0.831	0.640

**Table 7:** Highest-permittivity insulating candidates ($E_{g}\geq 4$ eV) from the corrected pool, with calibrated predictive standard deviations and the maximum Tanimoto similarity of each candidate to the training set.

Repeat Unit	ε	$\sigma_{\varepsilon }$	$E_{g}$ (eV)	$\sigma_{E_{g}}$	NN Sim.
CO–NH–CO–O	4.41	0.68	4.82	0.63	0.50
O–CO–NH–$C_{6}H_{4}$	4.27	0.67	4.09	0.50	0.67
CS–O–${\mathrm{CH}}_{2}$–NH	4.15	0.69	4.00	0.54	0.30
NH–CS–O–${\mathrm{CH}}_{2}$	4.12	0.68	4.27	0.52	0.50
CO–CO–${\mathrm{CH}}_{2}$–NH	4.11	0.68	4.52	0.59	0.32
CO–${\mathrm{CH}}_{2}$–CO–NH	4.11	0.68	4.83	0.58	0.47
NH–CO–${\mathrm{CH}}_{2}$–NH	4.06	0.70	5.13	0.62	0.33
O–${\mathrm{CH}}_{2}$–O–CS	4.00	0.68	4.09	0.58	0.29
CO–${\mathrm{CH}}_{2}$–NH–CO	4.00	0.68	4.47	0.70	0.50
CO–CO–${\mathrm{CH}}_{2}$–O	3.99	0.67	4.80	0.65	0.35

**Table 8:** Sensitivity of the screening outcome to the bandgap threshold. “Point” applies the criterion to the predicted gap; “uncertainty-aware” requires $E_{g}-1.96\;\sigma_{\mathrm{tot}}\geq E_{g}^{\min}$.

$E_{g}^{\min}$ (eV)	Nominated (Point)	Nominated (Uncertainty-Aware)	Max Predicted ε
3.0	246	63	5.53
3.5	157	32	4.68
4.0	65	19	4.41
4.5	34	9	4.41
5.0	21	4	4.06

**Table 9:** Named polymers contained in the four-block space: DFT reference values from the dataset [5] against out-of-fold machine learning predictions. $\varepsilon_{\mathrm{elec}}$ and $\varepsilon_{\mathrm{ion}}$ are the DFT electronic and ionic components of ε. Bold marks the two summary rows (mean absolute error and Spearman rho over the eleven polymers).

Polymer	Block Sequence	$\varepsilon_{\mathrm{DFT}}$	$\varepsilon_{\mathrm{ML}}$	$\varepsilon_{\mathrm{elec}}$	$\varepsilon_{\mathrm{ion}}$	$E_{g}^{\mathrm{DFT}}$	$E_{g}^{\mathrm{ML}}$
Polyethylene (PE)	${\mathrm{CH}}_{2}$-${\mathrm{CH}}_{2}$-${\mathrm{CH}}_{2}$-${\mathrm{CH}}_{2}$	2.61	3.00	2.59	0.02	8.61	5.81
Aliphatic polyether	${\mathrm{CH}}_{2}$-${\mathrm{CH}}_{2}$-${\mathrm{CH}}_{2}$-O	2.84	3.14	2.53	0.31	7.19	6.70
Polyoxymethylene (POM)	${\mathrm{CH}}_{2}$-O-${\mathrm{CH}}_{2}$-O	3.59	3.36	2.58	1.01	7.13	6.23
Poly(*p*-xylylene)	${\mathrm{CH}}_{2}$-$C_{6}H_{4}$-${\mathrm{CH}}_{2}$-$C_{6}H_{4}$	3.21	3.36	3.12	0.09	4.78	4.15
Aromatic polyester	${\mathrm{CH}}_{2}$-CO-O-$C_{6}H_{4}$	3.25	3.58	2.78	0.47	4.91	4.39
Poly(phenylene oxide)	$C_{6}H_{4}$-O-$C_{6}H_{4}$-O	3.66	3.59	3.27	0.39	3.91	4.49
Poly(ether ketone)	CO-$C_{6}H_{4}$-O-$C_{6}H_{4}$	3.89	4.01	3.27	0.62	3.45	3.44
Aromatic polyurea	NH-CO-NH-$C_{6}H_{4}$	4.22	4.71	3.42	0.80	3.61	3.77
Polyurea	NH-CO-NH-CO	4.75	4.74	3.12	1.63	4.17	4.78
Poly(*p*-phenylene) (PPP)	$C_{6}H_{4}\times 4$	4.18	4.54	4.11	0.07	2.75	3.28
Polythiophene (PT)	$C_{4}H_{2}$S × 4	9.03	8.55	8.80	0.23	1.02	1.18
**MAE over the eleven polymers**		0.27				0.67 eV	
**Spearman ρ (DFT vs. ML)**		0.97				0.90	

## Data Availability

Publicly available datasets were analyzed in this study. The four-block polymer dataset was originally reported by Mannodi-Kanakkithodi et al. [5] and is available at https://doi.org/10.1038/srep20952 (mirror: https://github.com/zhangtia/ML-Dielectric-Polymers (accessed on 5 August 2026)). The analysis code that reproduces all results is provided as Supplementary Materials.

## Supplementary Materials

The following supporting information can be downloaded at https://www.mdpi.com/article/10.3390/polym18182182/s1.
